# The American Pocket Medical Dictionary

**Published:** 1903-11

**Authors:** 


THE AMERICAN POCKET MEDICAL DICTIONARY.—Edited by W. A. Newman Dorland, M. D., Assistant Obstetrician to the Hospital of the University of Pennsylvania. Containing the pronunciation and definition of the principal words used in medicine and kindred sciences, with 566 pages, and sixty-four extensive tables. Philadelphia, New York, London: W. B. Saunders & Company. 1903. Flexible leather, with gold edges; $1.00 net; with thumb index, $1.25 net.

This little dictionary, now in its fourth edition, is indeed a very valuable little work containing, as it does, the essential facts associated with nearly every word in medicine and surgery.

This edition has been thoroughly revised and a great many of the newest terms appearing in recent medical literature are included. We take pleasure in recommending the work to students and physicians. J. M. L.

